# Supplementary material for: Non-Coding, RNAPII-Dependent Transcription at the Promoters of rRNA Genes Regulates Their Chromatin State in S. cerevisiae
Source: Noncoding RNA. 2021 Jul 11;7(3):41. doi: 10.3390/ncrna7030041 (PMC8293398; doi:10.3390/ncrna7030041)
Supplement: Supplementary file 1 [file ncrna-07-00041-s001.zip › ncrna-1262953-supplementary.pdf]

non-coding RNA

Supplementary Material

Non-Coding, RNAPII-Dependent Transcription at the Promoters of rRNA Genes Regulates Their Chromatin State in *S. cerevisiae*

Emma Lesage †, Jorge Perez-Fernandez †,‡, Sophie Queille, Christophe Dez, Olivier Gadal * and Marta Kwapisz *

Laboratoire de Biologie Moléculaire, Cellulaire et du Développement (MCD), Centre de Biologie Intégrative (CBI), Université de Toulouse, CNRS, UPS, 31062 Toulouse, France; emma.lesage-chevillon@univ-tlse3.fr (E.L.); jorge.perezfernandez@ujaen.es (J.P.-F.); sophie.queille@univ-tlse3.fr (S.Q.); christophe.dez@univ-tlse3.fr (C.D.)

* Correspondence: olivier.gadal@univ-tlse3.fr (O.G.); marta.kwapisz@univ-tlse3.fr (M.K.); Tel.: +33-(0)5-6133-5939 (O.G.)

† These authors contributed equally.

‡ Present address: Departamento de Biología Experimental, Facultad de Ciencias Experimentales, Universidad de Jaén, Paraje de las Lagunillas, s/n, 23071 Jaén, Spain.

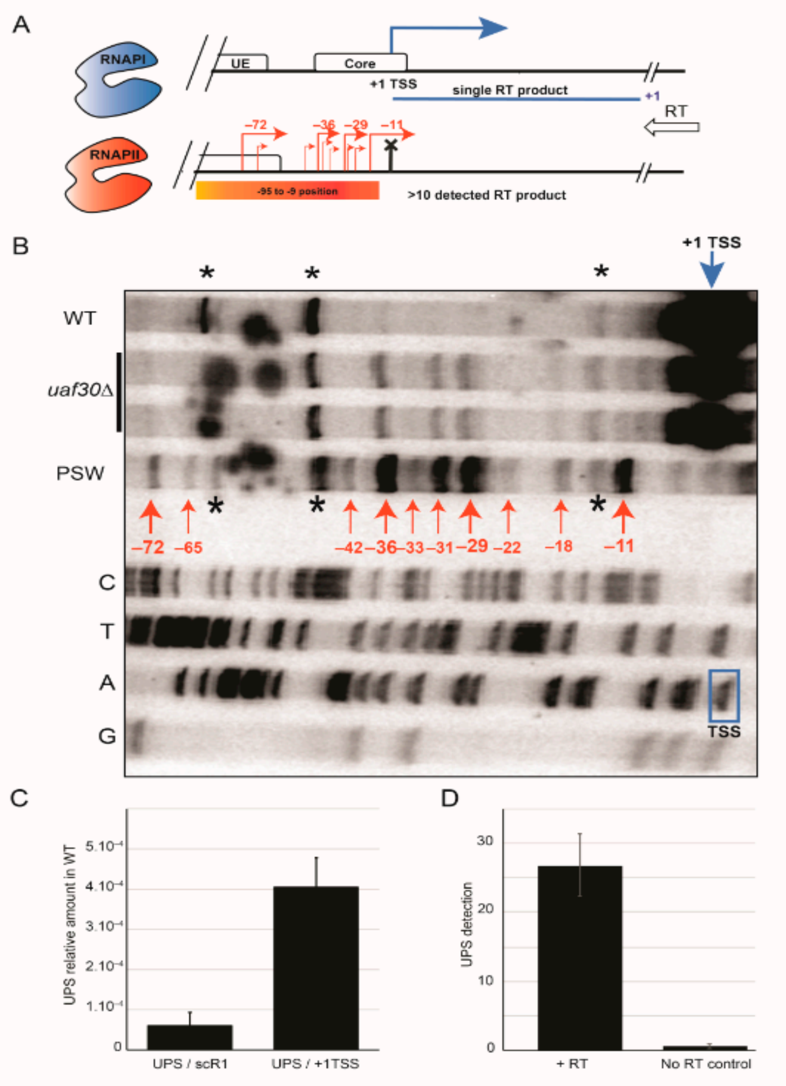

Figure S1. UPS lncRNAs are transcribed from the IGS2 region in rDNA. **A.** Schematic representation of the 35S promoter region composed of UE (Upstream Element) and Core promoter. RT forward

primer was used for reverse transcription. Blue arrows represent +1 TSS. Red arrows represent position of 5′ of UPS rRNA. **B.** Primer extension reaction with radiolabelled RT primer was resolved in 10% denaturating acrylamide gel. +1 TSS is marked by blue arrows and UPS by red ones. Star (*) represents a non-specific RT product. Sequencing reaction was used as molecular weight **C.** Quantification of UPS in wild type strains. UPS were quantified using RT primer for reverse transcription (1 µg of total RNA) relative to *scR1* and +1 TSS. Error bars correspond to the standard deviation of three independent cultures. **D.** 1 µg of total RNA was used with or without reverse transcription (no RT), showing that UPS can be detected about 50-fold over background.

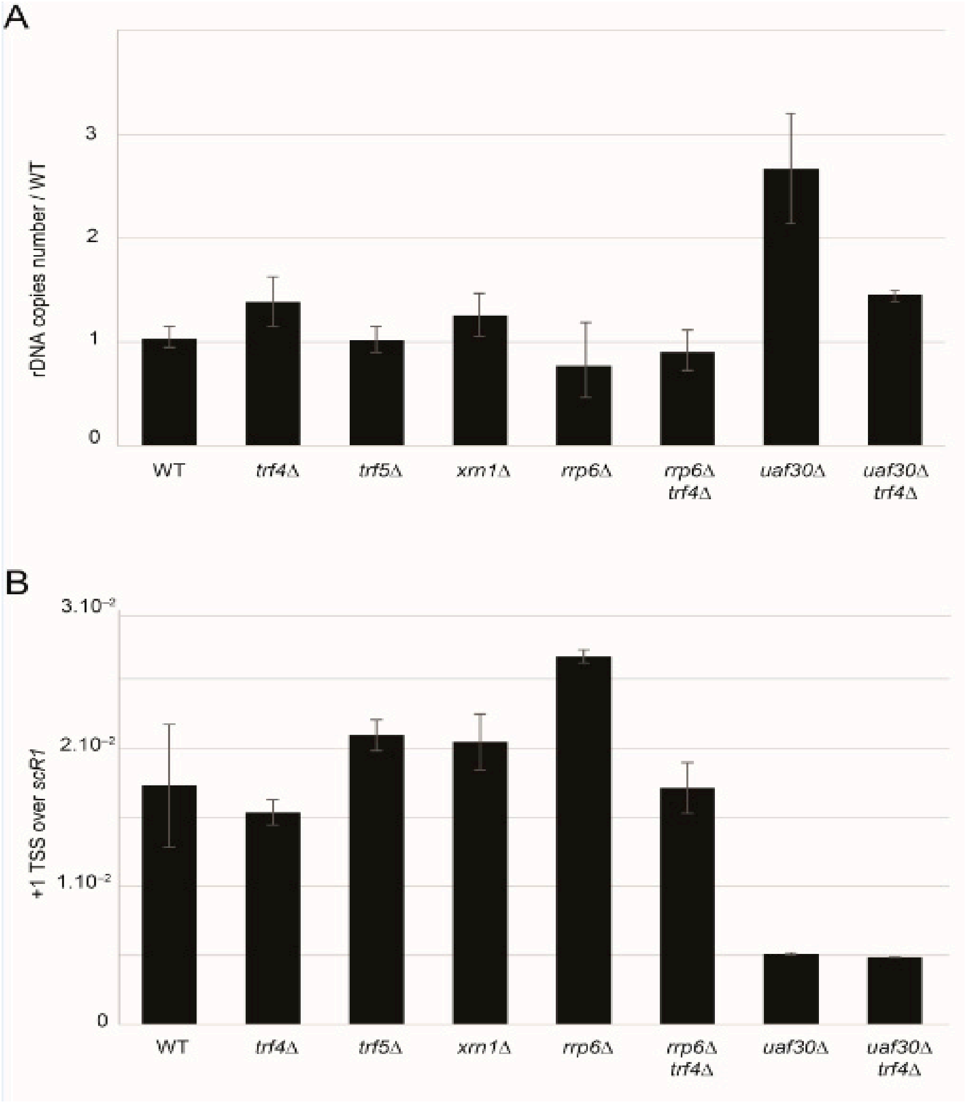

Figure S2. rDNA copy number and +1 TSS rRNA accumulation in various mutants. **A.** Quantification of rDNA copy number was performed using qPCR and are shown relative to WT control (BY4741). **B.** Quantification of +1 TSS in various mutants relative to *scR1* accumulation. Error bars correspond to the standard deviation of three independent cultures.

Table S1. Strains used in this study.

Name	Genotype	Source / reference
WT BY4741	*MATa, his3Δ, leu2Δ0, met15Δ0, ura3Δ0*	Euroscarf
WT BY4742	*MATα, his3Δ1, leu2Δ0, lys2Δ0, ura3Δ0*	Euroscarf
WT BY4743	*MATa/α, his3Δ1/his3Δ1 leu2Δ0/leu2Δ0 LYS2/lys2Δ0 met15Δ0/MET15 ura3Δ0/ura3Δ0*	Euroscarf
rrp6Δ	*MATα, lys2, ura3, his3, leu2, rrp6::KAN-MX4*	Euroscarf
trf4Δ	*MATα, ura3, his3, leu2, trf4::KAN-MX4*	Euroscarf
trf5Δ	*MATα, lys2, ura3, his3, leu2, trf5::KAN-MX4*	Euroscarf
rrp6Δ trf4Δ	*MATα, met15, lys2, rrp6::NAT-MX, trf4::KAN-MX4*	This study
uaf30Δ	*MATa, his3Δ1, leu2Δ0, lys2Δ0, ura3Δ0, met15Δ0, uaf30::KAN-MX4*	This study
xrn1Δ	*MATa, his3Δ1, leu2Δ1, ura3Δ0 met15Δ0, xrn1::KAN-MX4*	Euroscarf
rat1-1	*MATa, his3Δ1, leu2Δ1, ura3Δ0 met15Δ0, rat1-1::NAT-MX*	[1]
rpb4Δ	*MATa, his3Δ1, leu2Δ1, ura3Δ0 met15Δ0, rpb4::KAN-MX4*	Euroscarf
uaf30Δ trf4Δ	*MATa, his3Δ1, leu2Δ0, lys2Δ0, ura3Δ0, met15Δ0, uaf30::KAN-MX4 trf4::NAT-MX*	This study
rpa49Δ	*MATα, his3Δ1, leu2Δ0, ura3Δ0, met15Δ0, lys2Δ0 rpa49::alpha NAT-MX*	This study
pMET3-RAT1	*MATa, ade2-1 (ochre), can1-100 (ochre), his3-11,15, leu2-3,112, trp1-1 (ambre), ura3-1 HIS3::pMET3-RAT1*	[2]
pMET3-RAT1 rrp6Δ	*MATa, ade2-1 (ochre), can1-100 (ochre), his3-11,15, leu2-3,112, trp1-1 (ambre), ura3-1 HIS3::pMET3-RAT1, rrp6::KAN-MX4*	This study
WT YPH499	*MATa, ura3-52, lys2-801_amber, ade2-101_ochre, trp1-Δ63, his3-Δ200, leu2-Δ1*	[3]
rpb1-1	*MATa, leu2-Δ1, ura3-52, trp1-Δ63, lys2-801, his3-Δ200, rpb1-1 (rpb1-G1437D)*	[4]
rDNA 190C	*MATa, leu 2-3, 112 ura3-1, his3-11, trp1-1, ade2-1, can1-100, fob1::HIS3 rDNA copy number 190*	[5]
rDNA 25C	*MATa, leu 2-3, 112 ura3-1, his3-11, trp1-1, ade2-1, can1-100, fob1::HIS3 rDNA copy number 25*	[5]
PSW	*MATa, rpa135::LEU2, rrn9::HIS3, ade2-1, ura3-1, trp1-1, leu2-3, 112, his3-11, can1-100*	[6]

Table S2. Primers used in this study.

Name	Sequence 5′-3′
RT (35S rRNA) - R	ACACGCTGTATAGAGACTAGGC
A (35S rRNA) - R	GACTCTCTCCACCGTTTGACG
B (35S rRNA) - F	AAAGCAGTTGAAGACAAGTTCGAA
C (UPS) - R	ACGACAAGCCTACTCGAATTCGT
D (UPS) - F	GAGGAAAAGTAGTTGGGAGGTAC
sCR1 - F	GGCTGTAATGGCTTTCTGG
sCR1 - R	GTGCGGAATAGAGAACTATCC
pGAL7 - F	CGAACAGTAGCTGATCTCAG
pGAL7 - R	ATTTAACCAAATGGTGAAGGAGGACCTCGC
PMA1 - F	CCTCTTCATCATCCTCTTCAGC
PMA1 - R	AGCGTCATCGTAAGTCTTAGCAG
PCR rDNA - F	CTTGTCTCAAAGATTAAGCCATGC
PCR rDNA - R	ACCACAGTTATACCATGTAGTAAAGGAACT

References

1. Iglesias, N.; Redon, S.; Pfeiffer, V.; Dees, M.; Lingner, J.; Luke, B. Subtelomeric repetitive elements determine TERRA regulation by Rap1/Rif and Rap1/Sir complexes in yeast. *EMBO Rep.* **2011**, *12*, 587–593, doi:10.1038/embor.2011.73.
2. El Hage, A.; Koper, M.; Kufel, J.; Tollervey, D. Efficient termination of transcription by RNA polymerase I requires the 5′ exonuclease Rat1 in yeast. *Genes Dev.* **2008**, *22*, 1069–1081, doi:10.1101/gad.463708.
3. Sikorski, R.S.; Hieter, P. A system of shuttle vectors and yeast host strains designed for efficient manipulation of DNA in Saccharomyces cerevisiae. *Genetics* **1989**, *122*, 19–27.
4. Briand, J.F.; Navarro, F.; Gadal, O.; Thuriaux, P. Cross talk between tRNA and rRNA synthesis in Saccharomyces cerevisiae. *Mol. Cell. Biol.* **2001**, *21*, 189–195, doi:10.1128/MCB.21.1.189-195.2001.
5. Machín, F.; Torres-Rosell, J.; De Piccoli, G.; Carballo, J.A.; Cha, R.S.; Jarmuz, A.; Aragón, L. Transcription of ribosomal genes can cause nondisjunction. *J. Cell Biol.* **2006**, *173*, 893–903, doi:10.1083/jcb.200511129.
6. Vu, L.; Siddiqi, I.; Lee, B.S.; Josaitis, C.A.; Nomura, M. RNA polymerase switch in transcription of yeast rDNA: Role of transcription factor UAF (upstream activation factor) in silencing rDNA transcription by RNA polymerase II. *Proc. Natl. Acad. Sci. USA* **1999**, *96*, 4390–4395, doi:10.1073/pnas.96.8.4390.
